# On Drugs

**DOI:** 10.1093/jmp/jhad035

**Published:** 2023-06-23

**Authors:** Sam Baron, Sara Linton, Maureen A O’Malley

**Affiliations:** Dianoia Institute of Philosophy, Australian Catholic University, Melbourne, Australia; Clinical Pharmacy, Royal Melbourne Hospital, Melbourne, Australia; History and Philosophy of Science, University of Sydney, Sydney, Australia

**Keywords:** *conceptual analysis*, *drug*, *monism*, *pharmacology*, *pluralism*

## Abstract

Despite their centrality to medicine, drugs are not easily defined. We introduce two desiderata for a basic definition of medical drugs. It should: (a) capture everything considered to be a drug in medical contexts and (b) rule out anything that is not considered to be a drug. After canvassing a range of options, we find that no single definition of drugs can satisfy both desiderata. We conclude with three responses to our exploration of the drug concept: maintain a monistic concept, or choose one of two pluralistic outcomes. Notably, the distinction between drugs and other substances is placed under pressure by the most plausible of the options available.

## I. INTRODUCTION

Medicine in both its current and historical forms is almost inconceivable without drugs. Virtually every aspect of medical care, from diagnosis to cure, involves the prescription and administration of drugs. But what exactly are drugs in the modern medical sense of the term? According to Benedetti, a pioneer of mechanism-based work on placebos, “defining a drug is an easy task" (2014, 329). A drug:

…is a molecule delivered to the body to produce a biological effect. Its mode of action is to alter one or more biochemical pathways, for instance, by binding to a receptor or by modifying the activity of an enzyme. (Benedetti, 2014, 329)

In contrast, our account shows that the task of defining medical drugs is far from easy. Not only is Benedetti’s definition too narrow, but there is no viable definition of what drugs are that includes all and only those substances that are considered to be drugs in modern medicine.

Our paper is thus a first salvo against a conceptually monistic view of medical drugs: the idea that there is a single set of necessary and sufficient conditions that defines the medical drug concept. Why target a monistic account of drugs? Why not simply assume a degree of conceptual pluralism from the outset? This would, after all, fit a growing trend within philosophy of science toward pluralism for a number of scientific concepts from a range of domains, including “concept” itself (e.g., Dupré, 1999; Stotz, Griffiths, and Knight, 2004; Weiskopf, 2009).

One reason we begin with a monistic view is that conceptual monism seems to underlie much of the thinking about drugs within medicine and biomedical science. This is certainly evinced in Benedetti’s confident assertion above of what drugs are, but he is not alone. Most pharmacology textbooks begin with a brief but broad definition of drugs (see Section III) and then quickly move on to the task of explaining how drugs work.

A second reason for focusing initially on a conceptually monistic view of drugs is that it could be true in the restricted medical context we investigate. If legal and recreational drug concepts were also part of our inquiry, then a pluralistic view of drugs might seem a reasonable starting point. Currently, however, there is no existing body of work in philosophy that strongly suggests a plurality of drug concepts operating within medicine. Indeed, there has been very little philosophical work on the nature of medical drugs, despite their central role in medical practice. A consideration of the drug concept needs to start somewhere, and investigating the case for conceptual monism is an efficient way to begin an overdue philosophical discussion of drugs.

Even if a singular account of drugs fails, which we ultimately show to be the case, there is value in starting our investigation from a monistic basis. An examination of this sort proceeds by offering an overarching definition of what drugs are and then testing it against various criteria of adequacy. The fault-lines of various definitions tried in this manner are likely to reveal the contours of any underlying plurality of concepts. The failure of any single drug definition to do what is required of it tells us something about where distinct but related drug concepts may lie in relation to one another.

In this paper, we canvass a range of options for defining drugs and show that none of them is adequate. We begin, in Section II, by saying a bit more about the motivations behind our project before identifying two desiderata for an adequate definition of the drug concept. In Section III, we look to pharmacology for guidance, and analyze existing pharmacological definitions to draw out a list of properties that might be used as a basis for developing a definition of the drug concept. We also briefly consider natural-kind definitions before, in Section IV, pressing on to look at functional-kind definitions. Finally, in Section V, we consider social-kind definitions. In all cases, we find no definition of drugs that satisfies the basic desiderata laid down in Section II. We conclude, in Section VI, by presenting three responses to our exploration of the drug concept: one revisionary monistic conception, and two pluralistic accounts of drugs. We suggest that on the two most plausible options, a distinction between drugs and some putatively non-drug substances is difficult to sustain.

## II. THREE MOTIVATIONS AND TWO DESIDERATA

Our conceptual analysis of drugs has three main motivations. First, drugs play a major role in treatment, which is probably the core and very rationale of medicine, and is thus important to any philosophy of medicine. While it is unlikely that treatment can be defined exclusively in terms of drugs, the administration of a drug for therapeutic purposes does appear to be at least a sufficient condition for medical treatment. A drug may also feature as part of a necessary condition on treatment. Medical treatments might simply consist of a certain set of therapies, one of which is drug therapy. This does not, of course, serve as anything like a deep account of treatment, and we are not offering a conceptual analysis of it. The point is merely that drug therapy is central to treatment in medicine, and so a clearer understanding of what drugs are is likely to help us better understand the philosophical nature of treatment.

Drugs, via their connection to treatment, may also play a role in clarifying notions of health and normal functioning, which are probably the most debated issues in philosophy of medicine (e.g., Ereshefsky, 2009; Griffiths and Matthewson, 2018). Consider a diabetic who takes insulin. When a person’s body produces insulin of its own accord in the course of normal functioning, the chemical is not considered to be a drug. However, once the body stops producing insulin and that chemical is subsequently administered to the body, at that point insulin is a drug. One sign of abnormal functioning, then, is the need to replace a chemical produced by the body with a drug. Drugs are capable of restoring normal function in specific circumstances. A similar point can be made about health. One marker of ill health is the chronic need to take a drug. Taking a drug is thus potentially sufficient to identify both abnormal functioning and ill health.

A definition of what drugs are can also help us shed light on certain pressing demarcation questions. This is our second motivation. One demarcation question concerns the relationship between drugs and placebos. Much of the literature on placebos, both within medicine and within philosophy, sets placebos up in fundamental opposition to drugs (Miller and Brody, 2011). One fairly natural line of thought is that the difference between drugs and placebos lies in their mode of action. While both placebos and drugs can bring about therapeutic benefits, the main causal action of a placebo is via our beliefs, whereas the main causal action of a drug is via some biochemical pathway. To put the point somewhat provocatively, as others have (e.g., Colloca and Benedetti, 2005), placebos alter the mind, whereas drugs alter the body.

Whether this distinction holds up to scrutiny depends on how drugs and placebos work. It has long been acknowledged how difficult it is to define the concept of a placebo and, in particular, to identify placebo effects (Kienle and Kiene, 1997; Miller and Brody, 2011). However, because placebos are set up in opposition to drugs, it may be the case that the conceptual difficulties surrounding the notion of a placebo are partly due to a corresponding lack of clarity surrounding drugs. Thinking about drugs might help us not only to answer the question of whether placebos are drugs, but also to understand what placebos *are*. Matters are complicated by the fact that many placebo chemicals (e.g., lactose, fatty acids) can produce physiological effects of the kind that are normally associated with drugs (Golumb et al., 2010). If the relevant chemical that is being administered as a placebo has the potential for a positive health outcome, and that outcome can be linked to the chemical action of the placebo, then the gap between placebos and drugs would seem to be narrowed (Benedetti, 2014).

Aside from posing a conceptual conundrum, the contrast between placebos and drugs influences how we think about the use of placebos as a control substance in clinical trials. Trials to establish the efficacy of a specific drug are often measured against the efficacy of a placebo. Clinicians often see such comparisons as a way of comparing the drug to an “inert” or “non-active” substance (Miller and Brody, 2011). There is a question, then, about whether placebos are an appropriate control substance. If they are drugs, and this has not been properly recognized, then the failure to view placebos as potential drugs, with chemical pathways of their own, may lead to misinterpretations of clinical trial results.

There is also the question of why placebos with demonstrated efficacy are not generally recommended as drug-based therapies alongside whatever drug they were tested against in clinical trials. Anti-depressants are a case in point. In a now-famous meta-analysis, Kirsch et al. (2002) argued that more than 80% of the effect of anti-depressants is a placebo effect. The authors concluded that anti-depressants are not, in fact, very effective. An alternative conclusion is that there are two categories of anti-depressant drug, one of which is more effective than the other (i.e., the placebo “drug”), but that line of thought is not taken very seriously. Drugs clearly occupy a position in medical treatment that placebos do not. A conceptual analysis of drugs can help us to understand this perceived difference between placebos and drugs and even challenge it (if, for instance, a conceptual distinction between drugs and placebos is ultimately unsustainable).

Another demarcation challenge arises for excipients. Excipients are constituents of medicines that aid in the delivery of a particular chemical to a physiological system (e.g., the material in an aspirin tablet, apart from the acetylsalicylic acid, that helps in the drug’s stability, gastric disintegration, and systemic absorption). As with placebos, there is a tendency to draw a distinction between excipients and drugs. The excipient is the “inert” or “inactive” component of a medicine, whereas the drug is the “active” component of the medicine (Jivraj, Martini, and Thomson, 2000).

There is, however, an increasing awareness that excipients are not as “inert” as previously thought. Excipients can and do interact with the rest of the chemical constituents of a medication to produce unwanted or unexpected effects (Pifferi and Restani, 2003; Haywood and Glass, 2011). Moreover, excipients can sometimes be used for therapeutic purposes in other contexts. For instance, stearic acid is a commonly used excipient because of its low toxicity. However, it has also been used therapeutically as a topical cream for burns in mice (Khalil et al., 2000). This raises a similar question to the one in the placebo case, namely: are excipients drugs? If the answer is “yes,” then this has clear implications for how to view the supposedly inert substances that are coupled with drugs for the purposes of administration. If the answer is “no,” then it would be useful to know exactly why.

Finally, determining what drugs are would help to distinguish pharmacology from other sciences. Pharmacology is supposed to be the distinctive science of drugs. However, without an account of what drugs are, it is difficult to differentiate pharmacology from the broader study of chemical interactions within biological organisms (biochemistry) or even from the study of chemicals (chemistry). Such a distinction would also shed light on certain normative questions: why should we apportion funding and resources to pharmacology per se over biochemistry or chemistry more broadly?

To frame our investigation into all these questions, we suggest two desiderata for an adequate definition of the drug concept. Although there may be other criteria that could be included, satisfying these two desiderata is a condition on the minimal adequacy of a definition. We thus offer these desiderata not as a comprehensive guide to what the drug concept should be, but rather as a starting point for investigating that concept from an initially monistic outlook.

First, a definition of medical drugs should capture everything that is considered to be a drug in medical contexts. It should not be the case, for example, that under a candidate definition paracetamol is in whereas antacids are out. If substances commonly taken to be medical drugs are excluded by any supposedly unifying definition, the consequences of that exclusion should be justifiable and acceptable to pharmacological and medical practice. Likewise, if the definition brings in substances that are normally excluded, their inclusion should be similarly justifiable and acceptable.

How do we determine whether a definition of drugs covers the relevant substances? This can be done by consulting a comprehensive drug compendium, which lists substances that are used medically. A prominent example is the drug bank compiled by the government-funded Canadian Institutes for Health Research (DrugBank Online).^1^ This list contains over 13,000 drug entries, which are searchable by molecular structure, therapeutic use, and countries where the drug is approved or actively used for medical purposes.^2^ Ideally, an overarching drug concept would be applicable to everything on this list.

Second, a definition of what medical drugs are should capture only those things that are considered to be drugs in medical contexts. They should thus not include anything that is not considered to be a drug. Insofar as there is any consensus on how to define drugs, it appears to be that food and nutrients are always deemed not to be drugs (Dale and Haylett, 2009; Ritter et al., 2020). An adequate definition of what drugs are should thus not allow food or nutrients to qualify. Relatedly, it should not follow from a drug concept that eating food is a way of taking drugs, except when drugs are added to food. Ideally, criteria for dealing with food will illuminate other supposed non-drug substances currently categorized as placebos and excipients.

## III. PHARMACOLOGICAL DEFINITIONS

A basic definition of medical drugs should thus include all the substances taken to be medical drugs and exclude all the substances that are not. An obvious place to begin our search for an overarching definition of the drug concept is pharmacology. We might even hope for some sort of pharmacological law that underpins the designation of every substance that counts as a drug. Unfortunately, when we look to pharmacology textbooks, we find very little agreement over what drugs are, even though pharmacologists recognize the importance of giving a clear and encompassing definition. From an analysis of pharmacological textbooks, we collated 24 explicit definitions of the drug concept.^3^ From these definitions, we can identify 10 components that are used to define drugs, either in combination with one another or on their own:

(1) Drugs are chemicals.
(2) Drugs affect biological or physiological function.
(3) Drugs have a specific manner of effect (i.e., through chemical action by interaction with specific molecular components, thus modifying the response of a cell or tissue to its environment).
(4) Drugs are used for the diagnosis or treatment of disease.
(5) Drugs are administered.
(6) Drugs extend life.
(7) Drugs alleviate pain or suffering.
(8) A drug is a constituent of a medicine.
(9) A drug is not a food or nutrient.
(10) A drug is or could be listed in a pharmacopeia.

A quick glance at these components reveals two things. First, definitions of drugs are surprisingly varied. Current definitions range from the exceedingly general definition of a drug as “a chemical that affects living tissues” (Bryant, Knights, and Salerno, 2011, 2–3) to the more specific definition of drugs in terms of “molecules that interact with specific molecular components of an organism to cause biochemical and physiologic changes within that organism” (Alenghat and Golan, 2017, 2). There is also a detectable cleavage in existing definitions between whether emphasis is placed on the biochemical or on the social features of drugs. Compare the above two definitions with the definition below that emphasizes therapeutic use:

Any substance or combination of substances presented as having properties for treating or preventing disease in human beings; Any substance or combination of substances which may be used in, or administered to, human beings, either with a view to restoring, correcting or modifying physiological functions by exerting a pharmacological, immunological or metabolic action, or to making a medical diagnosis. (Directive 2001/83/EC of the European Parliament, as cited in MRHA [2020])

Another notable feature of existing drug definitions is the tendency to try and rule out food by fiat. This not only speaks to the importance of identifying a concept that achieves the desired contrast with food, but also to the difficulty with which this contrast may be achieved in a principled way. For example:

A drug can be defined as a chemical substance of known structure, *other than a nutrient or an essential dietary ingredient*, which, when administered to a living organism, produces a biological effect (our emphasis; Ritter et al., 2020; ch. 1).

Although pharmacology fails to provide a single cohesive definition of what drugs are, it does provide a great deal of source material on which a viable definition might be based. We can thus use the 10 components identified above to examine how a definition of the drug concept might work.

A standard first move in the conceptual analysis of scientific concepts is to appeal to natural kinds. A traditional natural kind definition is one according to which there is some intrinsic physical property or cluster of intrinsic properties that are features of all drugs. Natural-kind definitions are worth briefly considering because definitions along these lines do appear to underwrite some thinking about drugs. They are, for example, found in some pharmacology textbooks. In general, however, definitions of this kind in the case of drugs tend to be either far too broad or far too narrow.

For instance, we could take the first component on the pharmacological list and just define drugs as chemicals. However, while it is plausibly a necessary condition on being a drug that it is a chemical, it can hardly be considered sufficient. There are clearly many chemicals that are not drugs (e.g., graphite, cobalt nitrate). Or, to take another example, suppose we say that all drugs are chemicals that share a certain structure. There is a range of molecular structures that drugs can have. These include inorganic salts (such as potassium chloride), small to moderate organic compounds (such as aspirin and other salicylates), and large complex proteins (such as insulin glargine). The trouble is that there is no common molecular structure that all drugs have in common, even though there are classes of drugs that share a particular molecular structure.^4^ Determining such structure helps with drug classification, but not for defining what drugs, in general, are.

There is one natural-kind-style definition that we are particularly keen to set aside. We mention it only because it seems to be a popular way to think about medical drugs outside the medical community. This definition appeals to a distinction between drugs and “natural” substances. It claims that the common property of all drugs is that they are manufactured or artificially produced chemicals and so are unnatural. Non-drugs—like food and nutrients—by contrast are natural substances, containing only chemicals that arise in nature. This definition fails, however, because the distinction between drugs and natural substances is open to numerous straightforward counterexamples. Acetylsalicylic acid (aspirin), for instance, can be derived from willow bark, and many other substances now considered drugs have similarly “natural” origins. Indeed, according to Bade, Chan, and Reynisson (2010), around 10% of drugs on the market are unaltered natural substances.

A much more plausible account of drugs focuses not on any intrinsic nature but, instead, on their functional biological properties: the specific manner in which they interact with biological organisms. In the next section, we consider functional-kind-based accounts of drugs that do precisely this.

## IV. FUNCTIONAL-KIND DEFINITIONS

Using the functions of drugs to define them means we need to focus not on what drugs *are*, intrinsically, but on what they *do*. In pharmacology, receptor theory has been the primary candidate for defining drugs in terms of their functional role. Historical discussion in the field reveals a quest for an underlying biological principle that not only distinguishes drugs from other chemicals but also explains and predicts how they work therapeutically in human bodies (e.g., Parascandola and Jasensky, 1974; Kenakin, 2004). Indeed, according to Rang, “Receptors lie … at the heart of pharmacology … they provide the basic framework and are [its] ‘Big Idea’” (2006, S9).

As receptors transformed during the twentieth century from unknown theoretical entities into specific biological molecules (historically proteins), their role in drug action seemed to illuminate the nature of drugs. The basic idea is that there are certain proteins inside physiological systems that are either activated to bring about a certain effect when chemicals bind to regions on proteins, or are blocked from bringing about an effect via the same process of binding. These receptor proteins are separated into four distinct classes of drug “targets,” which have binding sites that drugs act on: G-protein-coupled receptors, ion channels, enzymes, and transporter proteins (Ritter et al., 2020).

One option for defining drugs in terms of their functional role, then, is to define drugs in terms of receptor binding with respect to these four molecular targets.

Definition 1 (D1): Drugs are chemicals that bind to one of four receptor molecules found within biological organisms.

Now this definition has many exceptions: there are drugs that operate without binding to one of these four receptors (e.g., carboplatin, which interferes with DNA in cancer cells to inhibit growth; or bisphosphonates used in the treatment of osteoporosis).

These exceptions might indicate that we should try expanding the definition of what receptor molecules are. One broadened definition of a receptor is a “recognition molecule for a chemical mediator through which a response is transduced” (Ritter et al., 2020; ch. 2). The identification of several classes of receptor proteins thus offers a way to narrow the definition of drugs. Only certain chemicals bind to certain receptors. Not all chemicals are capable of binding to all or even some receptors in the relevant sense. A drug, from this perspective, is a chemical that binds to a particular receptor inside a physiological system (e.g., Neubig et al., 2003; Maehle, 2009).

Definition 2 (D2): Drugs are chemicals that bind to receptor proteins inside biological organisms.

D2 has the virtue of retaining receptor theory at the heart of the drug concept and thus capturing a widely held view about drugs in pharmacology, in which receptors are “the seminal concept that all classes of therapeutic agents produce their effects by acting as ‘magic bullets’ at discrete molecular targets [receptors]” (Winquist, Mullane, and Williams, 2014, 7).

Unfortunately, D2 is too narrow. There are drugs that function without binding to anything, either protein or non-protein. Concentrated electrolytes, such as potassium salts, are an example. They work by creating, restoring, or minimizing a charge gradient across a cell membrane. Osmotic laxatives work in a similar way. Antacids are also considered drugs but they alter the pH of the stomach via chemical reaction rather than by binding to anything.

Conversely, many processes in cells operate via binding mechanisms and yet the chemicals involved are not considered drugs. For instance, ATP, the energy conversion molecule in cells, binds to a variety of proteins in the everyday course of cellular activity and organismal function. Antibodies bind to receptors on foreign entities but are not drugs, at least not when they occur endogenously. Likewise, enzymes—the workhorses of the cell—bind to all kinds of chemical substrates in the process of carrying out a huge range of basic cellular processes. Despite the centrality of binding to their activity, these endogenously produced molecules are not drugs. As Rang puts the point:

Is there anything about drug–receptor interactions that distinguishes them, as a class, from other kinds of biochemical goings‐on, that might justify the use of the specific term “receptor theory”? Not obviously. (Rang, 2006, S14)

We could try expanding the definition of a drug even further, away from receptor theory and protein binding. We could focus only on the causal action of the drug in the most general terms. A drug is thus a chemical that induces a physiological change inside an organism (D3).

Definition 3 (D3): Drugs are chemicals that induce physiological changes inside organisms.

While D3 (or something like it) is a common enough definition to be found in pharmacology textbooks, it is far too broad. It easily includes essential nutrients, all of which have physiological effects on organisms. Food is a way of introducing chemicals—namely, nutrients—into an organism to bring about a physiological change.

Can we find a “sweet spot” between the too narrow definition of a drug as one that binds to receptors, and the too broad definition of a drug as something that induces a physiological change inside an organism? Put another way, can we give a functional-kind definition of what drugs are that achieves the desired contrast between drugs, on the one hand, and food and nutrients, on the other? One option might be to try and rule food out via an appeal to medicines, and in particular to the notion of drugs as the active component of medicines (D4).

Definition 4 (D4): Drugs are the active ingredients of medicines.

There are two problems with D4, however. First, it presumes some prior notion of a medicine that cleaves it apart from food. The notion of a medicine is no less in need of conceptual clarification than the notion of a drug itself. Second, at least some chemicals considered to be drugs do not straightforwardly contain an active ingredient. Consider, for instance, pro-drugs. Pro-drugs are substances that are introduced into the body but which have to be metabolized into another substance to carry out their designated activity (Rautio et al., 2008). It is the chemical outcome of the metabolic process that has the sought-after affect. Pro-drugs, according to the active ingredient view, cannot count as drugs because they do not contain the active ingredient, even though they ultimately help generate it. Of course, this depends on what “active ingredient” means. But that is precisely our point: it is not entirely clear how to specify the “active” part of “active ingredient” in such a manner that does not exclude various pro-drugs.

Another difficulty with D4 is that it is far from obvious that the drug is always the active ingredient rather than the entire medicine. Consider medical uses of cannabis. Cannabis contains at least 400 different chemical compounds, of which 61 are considered to be cannabinoids (National Center for Biotechnology Information, 2020). Of these 61, the two most well-known are Tetrahydrocannabinol (THC) and Cannabidiol (CBD). But many of the other cannabinoids are thought to modify the physiological effects of cannabis. It is not clear that there is a single active ingredient in cannabis, so much as a range of different chemicals having a systemic effect on an organism. Classifying drugs in terms of their active ingredients alone would cause considerable problems for cannabis and other such substances.

Another way to try and achieve the desired contrast between drugs and nutrients might be to try and define drugs in terms of a very specific type of causal pathway. Thus, one might accept that drugs and nutrients do the same thing in some cases, but the way in which drugs achieve their outcome is distinctive. Since we cannot use receptor theory to get the desired contrast, we would need some other way to differentiate the type of causal action. But hypothetically, at least, we could say:

Definition 5 (D5): A drug is a chemical with a specific type of causal action inside physiological systems.

Aside from leaving the causal action of drugs dangerously underspecified, D5 faces a potential counterexample in the form of pro-drugs. As already noted, the type of causal pathway that leads from a pro-drug towards its effect is very similar, and plausibly of the same type, as the causal pathway that leads from some foods to nutrition.

Indeed, for at least some pro-drugs, the particular metabolic process that it undergoes mirrors the catabolic process involving glucose that produces ATP. A central aspect of the production of ATP involves the phosphorylation of glucose, in which a phosphoryl group is added to the glucose molecule. Some pro-drugs achieve their effects via a similar process. A drug used to treat COVID-19, remdesivir, is actually a pro-drug that, when administered, gets transformed by the addition of new molecular components within the cell via phosphorylation. The resulting chemical compound, which is considered the “active metabolite,” then interacts with a molecular target in order to achieve the desired effect. While remdesivir does not produce ATP, it is difficult to discern a difference in the causal mechanism that transforms remdesivir and the causal mechanism that transforms glucose.

Perhaps another option for achieving the desired contrast between nutrients and drugs in terms of causal action would be to emphasize their different effects. For instance, we might base a definition of what drugs are on a prior distinction between sustaining life and improving health. Thus, a nutrient is a chemical that is needed to sustain the physiological processes that enable an organism to live, whereas a drug is a chemical that induces a physiological change that improves the health of an organism (but the organism does not require the chemical to live).

Definition 6 (D6): Drugs are chemicals inducing physiological changes inside organisms that improve the organism’s health rather than merely sustaining the organism’s life.

Now the problem with D6 is that drugs such as insulin are used to sustain life. This means that the contrast between sustaining life and improving health fails to differentiate drugs from nutrients in terms of their causal outcomes.

Perhaps, instead, we can understand drug outcomes with respect to a baseline of normal functioning (D6).

Definition 6 (D6): A drug is a chemical that restores or improves normal functioning.

The concept of “normal functioning” is a contested notion, but whichever account is used, problems arise. Arguably, the contraceptive pill (a drug), when used to prevent pregnancy, prevents “normal function” of the reproductive system. There are two ways of understanding normal function: statistical normality, and evolved normal function in which certain traits are normal because they are selected effects (Griffiths and Matthewson, 2018). For the first, an assessment is made of what is typical of the reference class (let us say, healthy women between 20 and 40 years old in Western societies). The large majority of these women can conceive and in the “normal” course of affairs would do so. Contraception ends up creating a new normality (not conceiving). Only in the sense of this new statistical fact (that 50% or more of the population are taking contraceptives) are contraceptive drugs “restoring (new) normal function.” But this appears to be vacuous.

The evolutionary case for normal function depends on populations and their reproductive capacities. Human beings and other organisms succeed evolutionarily when they leave more offspring. Evolution can thus be thought of as “rewarding” reproduction, which is a selected normal function. Contraception is in fact preventing the basic “normal” thing human beings have evolved to do: reproduce. Now of course contraception may enable better parental care of fewer offspring, and thus more evolutionary success, but it is not entirely clear that a standard evolutionary explanation can encompass the direct mechanism by which this is achieved (contraceptive drugs).

The normativity of “normal” might be what is preventing D6 from working. What if drug function could be tied to a law, and normativity thus left out of the picture? Pharmacology, it could be argued, supplies a range of pharmacological laws that specify how drugs function within biological systems. A definition could thus be provided in terms of these laws. One difficulty with this move is that the subject matter of pharmacology would have to be specified in order to delimit its laws. However, this specification requires first providing an account of what drugs are. One way to overcome this problem would be to adopt an account of drugs that accepts a dependency between drug concepts and laws. A definition along these lines can be stated as follows:^5^

Definition 7 (D7): X is a drug iff X features in a pharmacological law, where Y is a pharmacological law iff it relates drug concepts.

The basic idea is to define drugs and pharmacological laws mutually, rather than using one as a foundation for the other. The definition is circular, but not necessarily in an objectionable sense. Indeed, circularity of this kind may offer a way to bootstrap the analysis into better conceptual territory. It also allows for future updates in that new drugs might invoke existing laws or, in some cases, help establish new laws, and vice-versa.

One way of applying D7 is to start with a clear case of a drug. Any laws that apply to the drug immediately qualify as pharmacological laws in virtue of featuring drugs. We can then work back down from the laws and see what else the laws cover. Anything further that the laws cover is then also classed as a drug. The second way of applying D7 is to start with a clear case of a pharmacological law. Substances that are covered by the law immediately count as drugs. Ideally, through this back and forth, the analysis ends up with a set of substances that contains all and only those things considered to be drugs, along with a group of laws that operate on just those substances.

D7, if made to work, would certainly be a principled way in which to provide a functional analysis of drugs. Unfortunately, it seems to yield the wrong results. Suppose we start with the laws. The best candidates for pharmacological laws are the general principles that govern receptor theory. Such laws include the Hill-Langmuir equations and the Michaelis-Menten equation. Together, these equations form a quantitative model of receptor theory by describing the way in which enzymes bind to molecules over time (in the presence or absence of agonists and antagonists that can reverse the binding process) and how substances change in the presence of enzyme binding (Chou, 1976; Colquhoun, 2006). The problem is that these equations describe substances that are not drugs, and processes that are not generally associated with drug effects. The Hill-Langmuir equations, for instance, also apply to food and nutrients that are metabolized by enzymes in the gut, via a metabolic process that binds nutrient molecules to enzyme receptors.

Even if it were possible to specify or otherwise restrict the laws so that they do not apply to food and nutrients, there is also the issue of a great many drugs falling outside the scope of receptor theory. This problem could potentially be addressed by applying D7 in the second manner described above: by using drugs to specify the pharmacological laws. Thus, we start from those drugs that fall outside of receptor theory and work upward to additional pharmacological laws that operate on these substances. The trouble, however, is that there are drugs that when treated in this way will yield laws that encompass substances that are not drugs.

One example is mitotic inhibitors, which are a specific class of cancer drugs. Mitotic inhibitors work by inhibiting cell-division. Such substances are therapeutic because they target the fast-growing cells that give rise to cancerous masses. A general law could be devised that describes the way these substances inhibit cell growth. The trouble, however, is that there are many substances that will also satisfy the law and which are not considered drugs. As Matson and Stukenberg (2011, 143) note, “it is clear that many compounds that result in mitotic arrest are neither useful therapeutics nor even effective at killing cells.” For instance, benomyl, a carbamate, inhibits mitosis in mammalian cells (Gupta et al., 2004), but it is not a drug; it is a fungicide commonly used in agriculture. In general, chemotherapy drugs are difficult cases because they blur the line between being toxic enough to treat cancer and not so toxic that they ultimately lead to death. There will be many cases in which two very similar substances, with similar functions, lie on different sides of that divide.

In a general sense, then, the problem with functional definitions of drugs is that they are either too narrow, capturing at best receptor-based reactions, or they are too broad, capturing various kinds of metabolic processes or toxifying activities. It is difficult to strike the right balance between these competing demands in a principled way. Although we cannot rule out the possibility of defining drugs functionally via other means, it does not seem likely that focusing on their causal properties sets drugs apart from other kinds of chemicals (and nutrients in particular). Drugs do not seem, therefore, to be good candidates for a plausible account of functional kinds.

## V. SOCIAL-KIND DEFINITIONS

So far, we have argued that neither natural-kind nor functional-kind definitions yield a satisfying account of what drugs are. This brings us to another way of defining drugs. Within the ten components of drug definitions that we identified in Section III, there were distinctive social aspects. This suggests that being a drug is not a matter just of being a certain chemical, or of having a certain functional role but, rather, of being used for a particular socially defined purpose. In particular, drugs are administered by healthcare professionals for therapeutic ends (D8).

Definition 8 (D8): A drug is a chemical that is administered within a medical context with the aim of bringing about a positive health outcome.

D8 faces a familiar problem in that food is often prescribed in treatment contexts in order to improve health (e.g., the FOD-MAP diet for irritable bowel disease; low potassium diets for kidney disease). A particularly difficult case is total parenteral nutrition (TPN).^6^ TPN is a chemical infusion containing fat, glucose, amino acids, electrolytes, trace elements, vitamins, and minerals. It is given to people who are unable to absorb nutrients in the ordinary way, through their stomachs. It is usually administered continuously over 12–24 hours and can be administered as a short course for several days or on a long-term basis. The goal of administering TPN is clearly to bring about a positive health outcome. However, TPN contains the same nutrients as food (it is just that these nutrients are administered in a way that bypasses the stomach).

The prospects for refining D8 to handle TPN are not good. The definition has two aspects: the notion of administration and the notion of a positive health outcome. TPN is administered in the same manner as a range of intravenous drugs, and so there is no way to draw a distinction between TPN and other substances via administration alone. The notion of a positive health outcome is similarly difficult to refine. The health outcomes related to TPN are analogous to the desired outcomes for many drugs.

In light of the difficulty facing D8, one option might be to shift the emphasis from medical treatment to a different kind of social criterion, namely: regulatory regimes (D9).

Definition 9 (D9): A drug is a chemical that is subject to a specific set of healthcare regulations.

According to D9, a drug is any chemical that is regulated inside a medical context, such as a hospital, surgery, pharmacy, or medical practice *in a specific way*. What way is that? Presumably, whatever regulatory framework is imposed on drugs in particular as opposed to other aspects of medical care.

The problem with D9 is that there is no single set of healthcare regulations that might be used to give substance to that specification, especially when we consider relevant international regulations. The controversial treatment of acidified sodium chlorite, for example, is not recognized as a drug in the United States, but counts as an “orphan drug” in the EU for a motor neuron disease (EMA, 2013), even if evidence of its benefits is extremely limited.

There is also a deeper problem with regulatory definitions of what drugs are. If we simply define drugs as any chemicals that fall under a certain set of medical regulations, it becomes unclear how we determine what these regulations should apply to in the first place. One option might be just to include a list of chemicals within the relevant regulatory framework. But then there is a question of how we decide what to include on the list and what to exclude from the list. Again, it is not possible to call on a prior conception of what drugs are to decide what the list should contain, because a drug is just anything that is on the list.

A possible way forward might be to define drugs in normative terms. A drug would then be any chemical that *should* be regulated in a certain kind of way within healthcare contexts, rather than any chemical that is as a matter of fact on a list of regulated substances. Drugs could be defined as substances that should be regulated so as to minimize harm (D10).

Definition 10: A drug is a chemical such that its administration in a healthcare context ought to be regulated in order to minimize health-related harms.

D10 faces at least two challenges. First, it is unclear what “health” actually means. This is a much-contested notion in the philosophy of medicine (e.g., Ereshefsky, 2009), and so invoking a notion of health-related harm to define drugs may turn out to be more difficult than defining drugs. For instance, does health-related harm include harms to mental health? Presumably it should in order to include psychiatric drugs, but then we need not only an account of physical health, but of mental health. Any definition of mental health would need to be at least consistent with a definition of physical health, so we still need to know what health is. This is likely to bring us back to the manifold issues of notions like normal functioning that we outlined for D6. There is no agreed-upon notion of normal functioning that we can simply call on to fill out D10.

A second problem is that D10 still seems to be too broad. Once again, TPN is a case in point. TPN is strictly regulated in healthcare contexts, because there are health-related harms associated with the improper administration of this nutrient solution. For instance, TPN carries a risk of blood infection. While there is a risk of a blood infection with any intravenous solution, the length of time that TPN is administered and the high glucose content of the solution makes infection risk a serious concern. In addition, if TPN is improperly formulated by incorrectly balancing the electrolytes in the solution, there is a substantial risk of arrhythmia and seizure. There is therefore just as much need to manage the potential health-related harms associated with nutrient solutions as there is for any drug.

## VI. DISCUSSION

Our 10 definitions of drugs have not taken us any closer to a natural-kind-based definition, functional-kind-based definition, or even a social-kind-based definition of drugs. Any combination of intrinsic, functional, and social factors merely picks and chooses which elements of a basic list of properties to combine and emphasize, and each of those combinations runs into trouble. Although our discussion is by no means exhaustive, we do take it to cast doubt on the prospects for finding a robust definition of drugs that achieves the two desiderata identified in Section II.

One way forward in our conceptual understanding of drugs is to give up or otherwise modify one of these two desiderata in an effort to save a conceptually monistic view of drugs. Recall the two desiderata: (a) everything considered to be a drug should be captured, and (b) everything that is not considered to be a drug should be ruled out. The focus of (b), in particular, was on food and nutrients. A straightforward suggestion then is simply to give up on the contrast between medical drugs on the one hand, and food and nutrients on the other. While giving up the contrast with food will do little to resuscitate a functional or natural-kind definition of drugs, it does seem to bring two of the social-kind definitions back into play, specifically D8 and D10. So, for instance, we could adopt D8 and just concede that, within a medical context, food, and nutrients are drugs because they have a therapeutic benefit. This is not so extreme an outcome, especially once we take into account the comparability in physical action between many drug molecules and nutrients, specifically for nutrient solutions like TPN.

There are two important implications of adopting this response to the arguments presented here, however. First, if the administration of a drug is at least sufficient for medical treatment (as noted in Section II), then the administration of food and nutrients in a hospital counts as a dimension of medical treatment and care as well. This would not be limited to TPN. Simply feeding a patient in order to ensure a positive health outcome would plausibly fall under the broad rubric of medical treatment. This would lift the ethical stakes when it comes to how food is managed inside a hospital. In addition, insofar as all drugs ought to be regulated in some way within a healthcare setting, it follows that food and nutrients should be subject to a similar regulatory framework. Similar implications carry forward to notions of health and normal functioning. If chronic drug-taking is sufficient to define ill health and abnormal functioning, then patients on prescribed diets may well fall under these notions insofar as the food and nutrients prescribed satisfy the drug concept. This places an additional burden on conceptual analyses of health and disease.

The second implication relates to placebos and excipients. If the drug concept is defined via D8 or, indeed, via any of the social-kind definitions provided in Section VI, then we have clear answers to the two demarcation questions identified in Section II, namely: are placebos drugs? And are excipients drugs? The answer, in both cases, is “yes.” Both placebos and drugs can be administered with an aim of bringing about a therapeutic benefit (though, in the case of excipients, this may be an indirect relationship based on the need for a delivery mechanism for a certain chemical). Similarly, placebos and excipients can have ill effects, and so there is reason to regulate them in the manner that drugs are regulated. If nothing else, this suggests a reconceptualization of placebo-controlled trials. Such trials should not be thought of as experiments in which drugs are compared with non-drugs. These are trials in which one drug is compared with another.

A more positive corollary to these issues is whether “natural” remedies are substances distinct from drugs. D8 suggests they are drugs when they are used with the aim of bringing about a therapeutic benefit. This reclassification would have the virtue of no longer exempting such substances from the risk-benefit analyses to which drugs are subjected. If natural products, such as specific herbal remedies, are exempt from such analyses, this can produce harm when adverse effects outweigh benefit (Seef et al., 2015). In other cases, evidence-based treatments are sometimes replaced with “natural” products with no known benefit. A fairly common example is when chemotherapy or even cancer surgery is rejected in favor of herbal remedies, such as curcumin (an extract of turmeric), because of beliefs that such natural substances are safer than drugs (see Nelson et al., 2017). If so-called natural remedies are deemed to be drugs and subjected to standard risk-benefit analysis and oversight, the potential problems of using them would be highlighted and, perhaps, avoided.

For anyone wishing to avoid the implications of including food, placebos, excipients, and other substances as drugs, conceptual monism about drugs will have to be abandoned and pluralism conceded. Has our discussion revealed a plurality of distinct medical drug concepts? There does seem to be a more or less natural cleavage between substances that fall under a particular functional kind—namely, the kind specified by receptor theory and any associated pharmacological laws—and the rest, which share no particular physical or functional features in common. We can thus posit two distinct drug concepts instead of a single definition that captures all and only the substances considered to be drugs.

However, this “receptors and the rest” pluralism faces an important question: is there anything that these two distinct concepts share in common such that they are both concepts of drugs? What would that common ground be? One plausible answer is that both conceptualize drugs as chemicals that are used in a medical setting for a therapeutic benefit. Thus, both concepts have something like D8 as a necessary condition, although they differ in what else they build into the drug concept. The receptor-based concept of drugs adds to D8 the specification of a particular functional kind. The second, looser concept by contrast adds nothing. By being non-specific about causal action, it manages to capture the miscellany of other substances considered to be drugs. If this basic picture is right, however, then yet again it will be difficult to keep food, placebos, and excipients out of the second, non-receptor-based drug concept. For it seems that the necessary condition specified by D8 is also sufficient in this case.

Suppose, instead, that there is nothing that unifies the two drug concepts. It is unclear whether a form of pluralism this weak is deserving of the name. Rather, the situation looks more like a case of polysemy where, for historical reasons, people have come to use the term “drug” to pick out two quite different things within a medical context, with no unifying rationale. Still, pluralism of this kind is possibly the only way to avoid the implications discussed above. If we are forced toward this form of pluralism about drugs, however, then we may need to reconsider whether we should continue talking univocally about drugs in medicine. What we really have is a range of different substances and a single term for them, without any unifying criterion for the use of that term. In that case, it might be better for our conceptual economy to dispense with drug talk and speak directly in terms of the various substances to which the term “drug” is applied. This would still provide a kind of answer to our two demarcation questions concerning placebos and excipients, by showing that those questions are not conceptually viable in the first place.

Ultimately, our goal is not to select between the different options that result from our investigation. It is striking, though, that two of the options we present challenge the distinction between drugs and food/nutrients. We are reluctant to recommend collapsing this distinction, however, because any conceptual understanding of drugs is in its infancy, and—as we noted at the outset—outcomes unacceptable to pharmacologists, clinicians, and medical professionals will need a lot more justification. Furthermore, there may still be a way to rescue a monistic view of drugs despite our failure to find a viable candidate. Or, it could be that there is actually a way to develop a pluralistic account of drugs that manages to cleave them from food and other substances. We thus offer these options in the spirit of starting a broader conversation about drugs and their implications in the philosophy of medicine.

## Footnotes

1 For a recent overview of the database, see Wishart et al. (2018).

2 There are other lists that we could have used. In the United States, the Drug Enforcement Agency (DEA) administers a list of controlled substances, and a similar list is maintained in the United Kingdom. Both lists, however, are focused on substances that are abused (rather than medically administered). Indeed, the DEA’s list categorizes drugs in terms of their likelihood to be abused (rather than their medical implementation). In Australia, the Therapeutic Goods Administration (TGA) maintains a list that is much closer to the Canadian drug bank list in its scope. The TGA list, however, does not include many experimental drugs, which are part of the Canadian drug bank taxonomy.

3 These definitions are available from the authors as a supplementary file.

4 For example, penicillin antibiotics are a family of antibiotics with a core structure of a B-lactam ring joined to a thiazolidine ring; however, unique R side chains differentiate different penicillin members by contributing to different pharmacological and chemical properties.

5 We are grateful to an anonymous referee for suggesting a definition along these lines.

6 We thank an anonymous referee for suggesting TPN.
